# Effects of psilocybin-assisted therapy on treatment-resistant depression

**DOI:** 10.1192/j.eurpsy.2021.1836

**Published:** 2021-08-13

**Authors:** A. Fraga, D. Esteves-Sousa, J. Facucho-Oliveira, M. Albuquerque, M. Costa, P. Espada Dos Santos, N. Moura, A. Moutinho

**Affiliations:** 1 Psychiatry, Hospital de Cascais, Cascais, Portugal; 2 Psiquiatria E Saúde Mental, Hospital de Cascais Dr. José de Almeida, Alcabideche, Portugal; 3 Psychiatry Department, Ocidental Lisbon Hospital Center, Lisboa, Portugal

**Keywords:** Depression, Psilocybin, treatment-resistant depression

## Abstract

**Introduction:**

Major depressive disorder is a highly prevalent clinical condition, affecting more than 300 million individuals worldwide. About 1/3 of patients with MDD fail to achieve remission despite treatment with multiple antidepressants and are considered to have treatment-resistant depression (TRD). Novel antidepressants with rapid and sustained effects on mood and cognition could represent a breakthrough in the TRD and may potentially improve or save lives. Psilocybin, a classic hallucinogen, more commonly found in the Psilocybe mushrooms has a combined serotonergic and glutamatergic action. The preliminary evidence of antidepressant effects of psilocybin-assisted therapy indicates the potential of psilocybin-assisted therapy as a novel antidepressant intervention.

**Objectives:**

The authors elaborate a narrative literature review about the effects of Psilocybin-based therapy on patients diagnosed with treatment-resistant depression.

**Methods:**

PubMed database searched using the terms “Treatment-Resistant Depression AND Psilocybin” and targeting clinical trials. References of selected articles and review articles were also assessed.

**Results:**

2 articles evaluate psilocybin effects in 32 patients with TRD and showed that two doses of psilocybin alongside psychological support significantly reduces depressive symptoms. All patients presented some reduction in symptoms from baseline to one week after the second dose and reproduced immediate and substantial improvements in depression that ultimately could sustain up to 6 months.

**Conclusions:**

Psilocybin-assisted therapy is a very appealing new possibility in the treatment of depression. However, due to the small populations of the existing trials, future studies are needed to prove this positive association and to fully understand Psilocybin’s mechanisms of actions and effects.

**Disclosure:**

No significant relationships.

